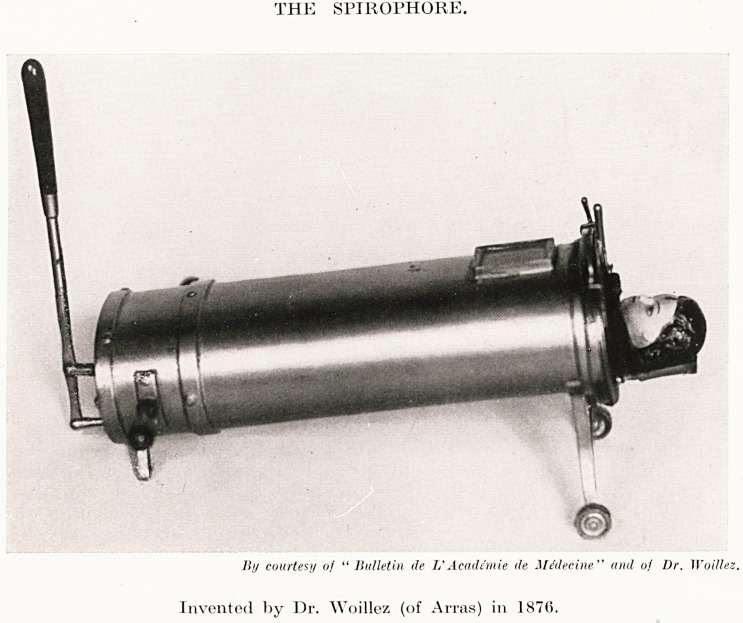# Drinker's "Iron Lung" and Other Artificial Respirators

**Published:** 1938

**Authors:** J. A. Nixon

**Affiliations:** Emeritus Professor of Medicine in the University of Bristol


### DRINKER'S "IRON LUNG" AND OTHER ARTIFICIAL RESPIRATORS.

BY

J. A. Nixon, C.M.G., M.D., F.R.C.P.,

*Emeritus Professor of Medicine in the University of Bristol.*

---

Lord Nuffield's splendid offer to supply the hospitals of the kingdom with artificial respirators or "iron-lungs" has aroused much interest and inquiry into the apparatus used for carrying out artificial respiration by mechanical methods.

One of the earliest types of artificial respirator was the "Pulmotor," which consisted of a face-piece such as is used for nitrous oxide anæsthesia, a long flexible rubber tube connecting the face-piece with a cylinder of compressed oxygen. Between the connecting or delivery tube and the cylinder of oxygen was a pair of small "concertina-type" bellows which were set in motion by releasing the oxygen from the cylinder. There was also a bag (as in Haldane's oxygen apparatus) which served to keep the oxygen pressure from rising too high in the delivery tube. Thus the oxygen on its way to the face kept the pump working, and this produced alternately a negative and positive pressure in the delivery tube provided the face-piece was closely applied to the face. This apparatus has been successfully used in mine-rescue work. When Rawlinson's army made its famous advance in front of Amiens on 8th August, 1918, a surprisingly large number of these, or some similar artificial respirators, were found in the captured German trenches. They had been kept there for the resuscitation of men overcome by carbon monoxide in mining operations or in dug-outs. One pulmotor was presented to the Bristol Royal Infirmary soon after the War. It cannot now be found! The suction effect on which this apparatus depends has been condemned as dangerous and ineffective by several authorities.

In the late nineteen twenties the high incidence of respiratory paralysis in an epidemic or series of epidemics of anterior poliomyetitis in the U.S.A. led Professor Drinker of Harvard University, after much experimental work, to design a chamber large enough to accommodate a grown male in which by variation of the surrounding air-pressure in the chamber artificial respiration could be maintained indefinitely by mechanical means. Professor Drinker's first models were made of wood, but he found steel to be more serviceable. The steel model was quickly named the "Iron Lung," and it was this model which Professor Drinker brought to England in 1930 and demonstrated to the profession. By the courtesy of Siebe, Gorman & Co. Ltd., the manufacturers of the apparatus, and Sir Robert Davis (managing director of the Company), we are enabled to give the following description and illustrations of Professor Drinker's apparatus as manufactured in this country by Siebe, Gorman & Co.

### THE DRINKER RESPIRATOR.

The Drinker respirator is a device for the prolonged administration of artificial respiration, and was designed by Dr. Phillip Drinker, of Harvard University. The Manual methods are not well adapted by prolonged use, and it is almost impossible by their means to produce and maintain adequate oxygen interchange in cases requiring long-time

### PLATE XXI

THE DRINKER RESPIRATOR.

*Photo. by courtesy of Messrs. Siebe, Gorman & Co. Ltd.*

*Photo. by courtesy of Messrs. Siebe, Gorman & Co. Ltd.*

Arm extensions for cases which require splinting.

THE BOTH CABINET RESPIRATOR.

PLATE XXII

*Photo. by courtesy of D. & J. Fowler Ltd.*

The Respirator in use.

administration. What is needed is a mechanical device which will be capable of working steadily over a long period of time, will permit control of the rate and depth of respiration, and will be capable of producing adequate artificial respiration without discomfort or harm to the patient. These requirements are satisfied by the Drinker respirator, an apparatus consisting of an enamelled steel chamber, which is large enough to accommodate a man 6 ft. 4 in. in height, and can also be used for a small child.

The patient lies on a sectional sponge-rubber mattress placed on a truck, which is attached to the lid of the chamber. The head and neck of the patient extend beyond the lid through a rubber collar, the head resting on a pad attached to the lid. The lid and truck of the chamber can be pulled in and out with ease to permit occasional examination of the patient, and these motions can be accomplished very rapidly. By means of an electrically-driven bellows the pressure inside the chamber is alternately reduced and restored to normal. Both speed and depth of respiration can be varied. In the event of failure of the electricity supply, means are provided for operating the machine by hand. Conscious patients can eat, drink and sleep in the respirator without stopping the mechanism. The head-rest and the upper part of the bed are adjustable for height. Tubular extensions can be provided, if required, to accommodate cases in which splinting of the arms is necessary. The apparatus can also be made to tilt in either direction.

In emergencies, and without pulling out the bed, enemas and rectal drips can be administered through the hand-holes provided in the side of the machine, and a rectal tube can be inserted by a person reaching in through one of the portholes.

The apparatus has been used successfully in cases of respiratory failure resulting from poliomyelitis, severe carbon monoxide poisoning, morphine and other drug poisons, alcoholic coma, hiccoughs, drowning, and, in the small size specially made for babies, numerous cases of asphyxia in the new-born.

The model which Lord Nuffield has selected is a modification of the "Drinker" iron lung designed by Mr. Both, and was manufactured by D. & J. Fowler Ltd. It is constructed in wood, and is known as the "Both Cabinet Respirator." Messrs. D. & J. Fowler have courteously supplied the following description of the apparatus and lent the block for its illustration:

BOTH CABINET RESPIRATOR.

The function of this machine is to operate the patient's lungs when for any reason, *e.g.* infantile paralysis, he has lost the power of operating them himself.

The machine consists of two parts, an air-tight cabinet in which the patient lies, and a mechanically operated bellows which reduces and increases the pressure in the cabinet. The two are connected by a flexible rubber tube.

The cabinet unit consists of a box made of laminated board which stands on a tripod to which is attached a tilting device enabling the cabinet to be tilted to any desired angle. Inside the box is a bed which can be easily slid in and out and the end of which forms one end of the box. The patient is placed on the bed with his head protruding through a hole in the end, the bed is then slid into the box which is made air-tight by the end being clamped on to the box by four quick-acting clamps. The joint is insulated with rubber mouldings, and a rubber band goes round the patient's neck so that the whole of his body is in an air-tight chamber while his head protuudes at one end.

The bellows unit consists of a $\frac{1}{2}$-h.p. electric motor which, through a set of reducing gears, drives a pair of bellows. These bellows draw the air out of the cabinet, thus allowing the patient's lungs to expand. The bellows then restore the pressure in the cabinet and empty the patient's lungs. The motor drives the gear-box by means of a rubber belt, and three different sized driving-wheels are fitted so that three different rates of pulsation can be obtained, 18, 24 and 32 per minute. Should the motor fail the drive to the bellows can be immediately disconnected and the bellows can be worked manually by a handle which projects through the top of the bellows unit, and is a permanent fixture.

The ventilation of the cabinet and the degree of pressure can be regulated by means of two valves, one placed on the cabinet, and the other on the top of the bellows unit, and a certain amount of attention can be given to the patient without opening the cabinet through two armholes which are fitted one on either side near the level of the patient's shoulders.

There is yet another mechanical respirator constructed on a different principle in which no box or cabinet is used, namely:

*Photo. by courtesy of Mr. R. W. Paul.*

Applicable to adults, adolescents and children without restriction of posture.

PLATE XXIII

THE SPIROPHORE.

*By courtesy of "Bulletin de L'Académie de Médecine" and of Dr. Woillez.*

Invented by Dr. Woillez (of Arras) in 1876.

PLATE XXIV

### THE BRAGG-PAUL PULSATOR.

It was originally devised by Sir William Bragg for the use of a friend suffering from progressive muscular atrophy as described in the *Lancet* by Dr. Phyllis M. T. Kerridge.[1]

Artificial respiration is maintained by the rhythmical application of pressure to the exterior of the chest around which a hollow rubber belt is fastened. Air at a definite pressure is forced into this belt by electrically driven bellows regulated to the appropriate respiration-rate. This pulsator is readily transportable, works quietly, and may be placed at any distance up to 8 feet from the patient, being connected to the air bag by a flexible hose-pipe. The pulsator is normally driven by electricity, it can also be worked in emergency by a hand-lever. About 50 Pulsators are already installed in hospitals in the United Kingdom. The apparatus is simply a mechanical air-belt substituted for the hands as employed in Schäfer's method of artificial respiration.

The method is one of applying positive pressure to the chest in contrast with the cabinet or box methods which depend upon negative pressure. Dr. Kerridge pointed out that positive pressure can be applied continuously for several years without ill effects. Further information can be had from Mr. R. W. Paul of 69 Addison Road, London, W. 14.

### THE SPIROPHORE.

The principle of the iron lung was first introduced in 1876 when Dr. Woillez, of Arras, a member of the French *L' Académie de Médecine*,[2] designed a mechanical respirator for the treatment of asphyxia under the name of the "Spirophore."

The apparatus consisted of a flattened metal cylinder large enough to admit the body of an adult. One end was capable of being hermetically closed by a lid, in the middle of which was an opening large enough to allow the patient's head to pass through, and furnished with a rubber sheet fixed at one part of

R

its circumference to the opening, and by another part capable of being fixed on the patient. At the opposite end of the cylinder a piston was fitted to move up and down in the cylinder by means of an outside handle. On the upper surface of the apparatus was a large window through which the interior could be seen. It was supported on small wheels and furnished with large handles so that it would be easily moved. The cylinder contained a sort of moveable stretcher which could be pulled in and out so as to place the patient in the most suitable position.

The method of use was to introduce the whole of the patient except the head and neck. The air-tight lid was closed and the rubber sheet was made to adhere carefully to the margin of the lower jaw and the back of the patient's neck. With the handle of the lever the piston was moved up and down, creating a alternate compression and exhaustion of the air in the interior of the cylinder. Through the window the movements of the thorax and abdomen were watched producing alternate dilation and contraction in a thoroughly physiological manner. Thus artificial respiration was completely and quietly maintained. The amount of air which could be inhaled into the lung by means of this apparatus was more than one litre. In the first model the piston worked in a small independent cylinder joined by large tubes to the main cylinder. In a special model made for new-born infants, a concertina bellows was adapted directly to the upper surface of the apparatus and was worked by hand.

The apparatus obtained a silver medal at an exhibition of life-saving apparatus held at Le Havre, soon after it had been exhibited at the Academy of Medicine in June, 1876. In spite of the advice of the manufacturer, Dr. Woillez always declined to patent the idea, being of the opinion that no obstacles should be placed in the way of the use of any apparatus designed for the saving of life.

REFERENCES.

[1] "Artificial Respiration for Two Years," *Lancet*, 1934, i. 786; "Artificial Respiration for Three and a Half Years," *Lancet*, 1936, i. 504.

[2] *Bulletin de l'Académie de Médecine*, 1938. 3° Série. Vol. 119, p. 82.

## Figures and Tables

**Figure f1:**
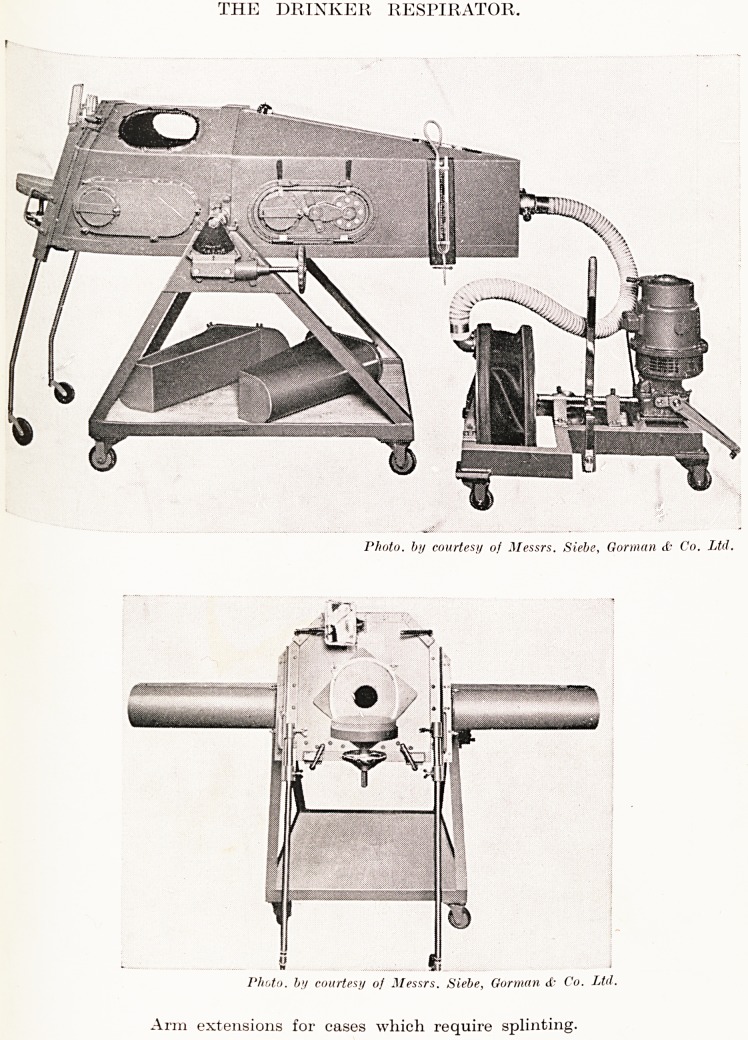


**Figure f2:**
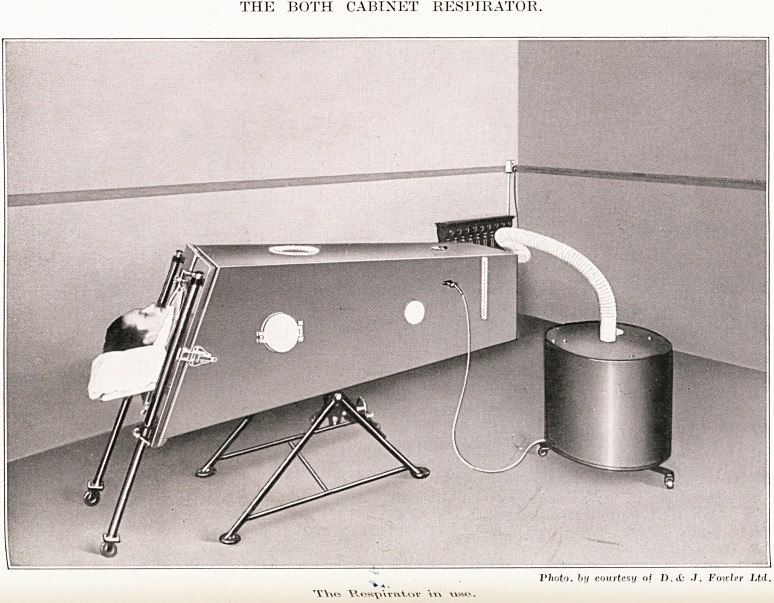


**Figure f3:**
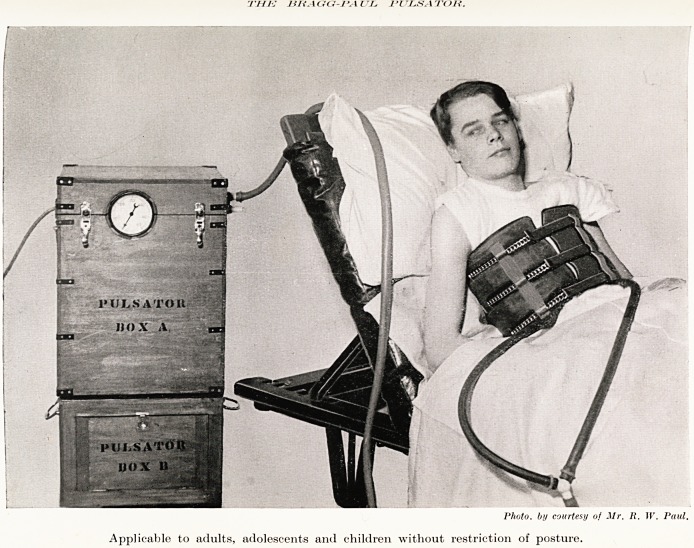


**Figure f4:**